# The Uncommon Active Site of D-Amino Acid Transaminase from *Haliscomenobacter hydrossis*: Biochemical and Structural Insights into the New Enzyme

**DOI:** 10.3390/molecules26165053

**Published:** 2021-08-20

**Authors:** Alina K. Bakunova, Alena Yu. Nikolaeva, Tatiana V. Rakitina, Tatiana Y. Isaikina, Maria G. Khrenova, Konstantin M. Boyko, Vladimir O. Popov, Ekaterina Yu. Bezsudnova

**Affiliations:** 1 Bach Institute of Biochemistry, Research Center of Biotechnology of the Russian Academy of Sciences, Leninsky Ave. 33, bld. 2, 119071 Moscow, Russia; a.bakunova@fbras.ru (A.K.B.); aishome@mail.ru (A.Y.N.); taniarakitina@yahoo.com (T.V.R.); inbimaldi@gmail.com (T.Y.I.); mkhrenova@lcc.chem.msu.ru (M.G.K.); boiko_konstantin@inbi.ras.ru (K.M.B.); vpopov@inbi.ras.ru (V.O.P.); 2 Shemyakin & Ovchinnikov Institute of Bioorganic Chemistry of the Russian Academy of Sciences, Miklukho-Maklaya Str. 16/10, 117997 Moscow, Russia; 3 Chemistry Department, Lomonosov Moscow State University, Leninskiye Gory 1/3, 119991 Moscow, Russia

**Keywords:** D-amino acid transaminase, enzyme catalysis, substrate specificity, X-ray analysis, arginine residues, sequence-structure-function relationships

## Abstract

Among industrially important pyridoxal-5’-phosphate (PLP)-dependent transaminases of fold type IV D-amino acid transaminases are the least studied. However, the development of cascade enzymatic processes, including the synthesis of D-amino acids, renewed interest in their study. Here, we describe the identification, biochemical and structural characterization of a new D-amino acid transaminase from *Haliscomenobacter hydrossis* (Halhy). The new enzyme is strictly specific towards D-amino acids and their keto analogs; it demonstrates one of the highest rates of transamination between D-glutamate and pyruvate. We obtained the crystal structure of the Halhy in the holo form with the protonated Schiff base formed by the K143 and the PLP. Structural analysis revealed a novel set of the active site residues that differ from the key residues forming the active sites of the previously studied D-amino acids transaminases. The active site of Halhy includes three arginine residues, one of which is unique among studied transaminases. We identified critical residues for the Halhy catalytic activity and suggested functions of the arginine residues based on the comparative structural analysis, mutagenesis, and molecular modeling simulations. We suggested a strong positive charge in the O-pocket and the unshaped P-pocket as a structural code for the D-amino acid specificity among transaminases of PLP fold type IV. Characteristics of Halhy complement our knowledge of the structural basis of substrate specificity of D-amino acid transaminases and the *sequence-structure-function* relationships in these enzymes.

## 1. Introduction

The superfamily of pyridoxal-5′-phosphate (PLP)-dependent transaminases (TAs; aminotransferases; EC 2.6.1.) is an excellent basis for studying *sequence-structure-function* relationships in enzymes: TAs exhibit a diversity of substrate specificity and activity using a uniform structural scaffold. TAs catalyze the stereoselective transfer of an amino group from an amino acid/amine to a keto acid/ketone/aldehyde, producing a new chiral amino acid/amine and a new keto compound. The transamination reaction proceeds via a double displacement reversible mechanism; one catalytic turnover includes two half-reactions with the conversion of covalently bound cofactor PLP to free pyridoxamine 5’-phosphate (PMP) and release of the first product and subsequent recovery of the PLP form of cofactor accompanying by transfer of the amino group to a keto substrate and the release of a new amino compound [1,2,3].

Transaminases belong to fold types I and IV of the PLP-dependent enzymes. The superfamily of PLP-dependent TAs of fold IV (a superfamily of D-amino acid aminotransferases) includes three canonical families: D-amino acid TAs (DAATs, EC 2.6.1.21), branched-chain L-amino acid TAs (BCATs, EC 2.6.1.42), and (*R*)-amine:pyruvate TAs (R-TAs, EC 2.6.1.X). Strict (*R*)-selectivity or (*S*)-selectivity of TAs of PLP fold type IV is implemented within geometrically similar active sites and results from the different amino acid compositions. Transaminases are usually homodimers, comprising two symmetrical active sites formed by the residues of both subunits. Characteristics of the residues in the active site determine the substrate specificity of TAs. Two specificity-determining sequence motifs, comprising residues of the active site, were identified to distinguish the substrate preferences of canonical TAs of PLP fold type IV [4,5,6]. Recently, TAs with a mixed activity or an expanded substrate specificity were identified; their specificity-determining sequence motifs differed from the motifs of canonical TAs [7,8,9].

DAATs are spread among bacteria as the main enzymes of the synthesis of D-glutamate, an essential component of the cell wall. In vivo, DAATs catalyze the transfer of the amino group from D-alanine to α-ketoglutarate, producing pyruvate and D-glutamate [10,11]. Only several DAATs are characterized today [12,13,14,15,16,17]. In vitro DAATs catalyze transamination between various D-amino acids and keto acids, including α-ketobyturate, indole-3-pyruvate, α-keto-γ-(methylthio)-butyrate, etc. [16]. However, α-ketoglutarate and D-alanine remain the best substrates for most of the characterized DAATs [12,13,15,16]. DAATs are prominent for the industrial synthesis of optically pure D-amino acids. In the last few decades, several applications of DAATs for the synthesis of unnatural amino acids for clinical purposes have been proposed [18,19,20,21,22].

Specificity-determining sequence motifs for DAATs were identified by the analysis of the active site of DAATs from the bacteria of genus *Bacillus*. These DAATs are considered canonical. As observed for DAAT from *Bacillus* sp.YM-1 (bsDAAT), three residues in the active site, Y31, R98*, and H100* (* indicates residues from the adjacent subunit of the functional dimer), determine the specificity towards D-amino acids [3,17]. These residues form a “carboxylate trap” for the binding α-carboxylic group of a substrate in a productive proD-position in the active site. However, the TA from *Curtobacterium pusillum* with an expanded substrate specificity [7], which catalyzes the synthesis of D-amino acids, using D-alanine or (*R*)-(+)-1-phenylethylamine (R-PEA) as amino donors, does not have a “carboxylate trap”. Lysine and arginine residues, nonequivalent to the R98* and H100* of bsDAAT, can bind α-carboxylic group in the active site. Thus, the question about the TA active site organization favoring efficient transamination of D-amino acids is still open and needs further consideration.

This study describes the identification, biochemical and structural characterization of a new D-amino acid transaminase from the bacterium *Haliscomenobacter hydrossis* (Halhy) [23]. Members of this species were isolated from freshwater lakes and ditch water. The optimal growth temperature and pH are 26 °C and 7.5, respectively. The new TA is a strict DAAT and features the new specificity-determining sequence motifs in the active site within a conserved scaffold of TAs of PLP fold type IV. The detailed structural analysis of the new DAAT and a comparison with the previously studied DAATs allowed us to suggest the structural basis of the DAAT specificity in a more general form than is accepted today for DAATs’ identification.

## 2. Results

### 2.1. Enzyme Identification, Expression, and Purification

Searching for sequences homologous to the sequence of TA from *C. pusillum* within the complete genome of *H. hydrossis* revealed a sequence of TA of PLP fold type IV (GenBank ID: WP_013764869.1), sharing sequence identity with TA from *C. pusillum* and bsDAAT 29.8% and 25.9%, respectively. The sequence of the putative transaminase contained unique specificity-determining sequence motifs, which differ from the sequence motifs of the canonical TAs of PLP fold type IV (Table 1).

Halhy was cloned and expressed in a soluble form in *E. coli* and purified to homogeneity (Figure S1). The molecular weight of the purified Halhy was approximately 62 kDa, as determined by gel filtration, indicating Halhy as a homodimer with a small amount of a tetramer in solution (Figure S2). The absorption spectrum of the holo form of Halhy had two characteristic peaks: one at 280 nm and the other at 416 nm (Figure S3). The latter indicated the formation of a Schiff base with PLP. No shift of the peak maximum was observed within the pH range 4–10 (Figure S4), showing the stability of the protonated form of the Schiff base up to pH 10. This feature is characteristic of TAs of PLP fold type IV [12,13,24].

### 2.2. Enzyme Activity, Substrate Specificity, and Enantioselectivity

**Scheme 1:**
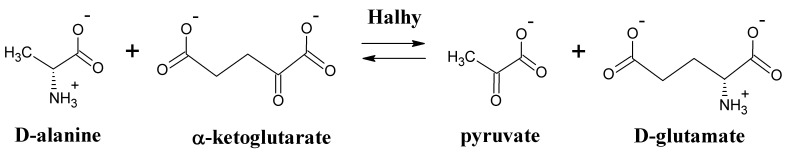
The enzymatic transamination reaction between D-alanine and α-ketoglutarate.

The analysis of the activity of Halhy in the overall transamination reaction with the benchmark substrates of TA of PLP fold type IV showed a strong preferences for converting D-amino acids and no activity towards L-amino acids and (*R*)-PEA (Table 2) (Scheme 1).

The amino acceptor spectrum of Halhy was examined in the overall reaction using D-glutamate as an amino donor (Table 3). Halhy showed the highest activity towards pyruvate and α-ketoglutarate, similar to the most characterized DAATs [12,13,15]. Notably, the Halhy activity in the overall reaction diminished with the increase of the length of the α-keto acid side chain.

The enantioselectivity of Halhy was evaluated in the synthesis of leucine and phenylalanine from keto analogs 4-methyl-2-oxovalerate or phenylpyruvate using D-glutamate as an amino donor. After 20 hours of the reaction at 30 °C, product yield achieved 98.5% and 95.6% in leucine and phenylalanine production, respectively. The enantiomeric excess of D-leucine and D-phenylalanine amounted to 99.4% and 99.3%, respectively (Supplementary, Figure S5).

### 2.3. Effect of Temperature and pH on Enzyme Activity and Stability

**Figure 1:**
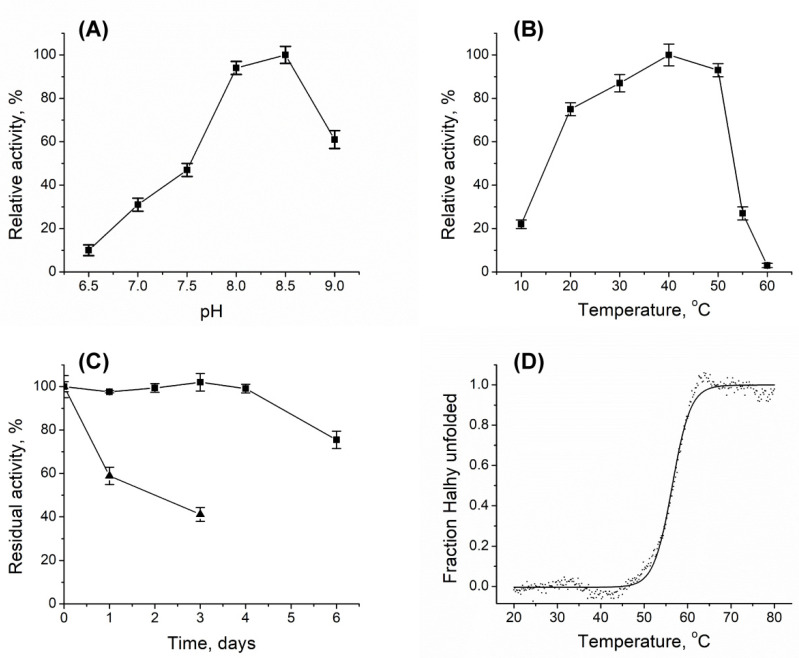
Kinetic and thermodynamic stability of WT Halhy. (**A**,**B**) the effects of pH and temperature on the overall transamination reaction with D-alanine and α-ketoglutarate; 100% corresponds to 27 ± 2 U/mg (**A**) and 50 ± 3 U/mg (**B**). (**C**) The residual activity of Halhy incubating at 40 °C (■) and 50 °C (▲): 100% corresponds to 50 ± 3 U/mg in the standard assay. Error bars represent standard deviation. (**D**) Thermal unfolding in K-phosphate buffer, pH 8.0.

The effect of pH and temperature on the enzyme activity was examined in the overall reaction with D-alanine and α-ketoglutarate. In a pH range of 6.5–9.0 (Figure 1A), Halhy showed maximum activity at pH 8.0–8.5. The optimum temperature of the transamination reaction was found to be 40 °C (Figure 1B). The stability of Halhy was examined at 40 and 50 °C: the activity of Halhy remained unchanged at 40 °C for four days (Figure 1C) and decreased 40% in a day at 50 °C. The *T*_0.5_ of the thermal unfolding of Halhy was 56.4 ± 0.1 °C (Figure 1D).

### 2.4. Steady-State Kinetic Parameters of the Overall Transamination Reactions 

The kinetic parameters of the overall transamination reactions catalyzed by Halhy are given in Table 4. As seen, Halhy showed a high turnover rate both in the direct reaction with D-alanine and α-ketoglutarate and in the reverse reaction with the D-glutamate and pyruvate (Scheme 1). The enzyme demonstrated the highest specificity towards α-ketoglutarate and pyruvate. Among the amino donors, the best substrate was D-glutamate.

### 2.5. The Overall Structure of Halhy

**Figure 2:**
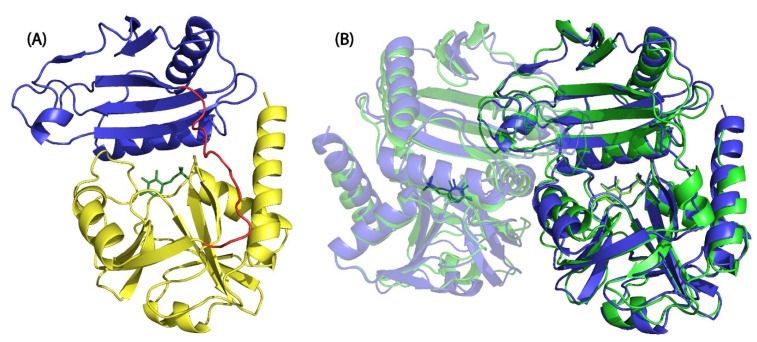
(**A**) The subunit of Halhy. The small domain is colored blue, the large domain is colored yellow, and the interdomain loop is colored red. The PLP molecule is shown as green sticks. (**B**) The active dimer of Halhy (blue) is superposed with the dimer of bsDAAT (green, PDB ID: 1DAA); the adjusted subunits of both structures are shown transparently.

The structure of WT Halhy in the holo form with a covalently bound PLP was determined to 2.0 Å resolution and deposited with PDB ID 7P7X. The subunit of Halhy has a structural organization typical of PLP-dependent TAs of fold type IV and is similar to the subunits of the known DAATs [4,17]. The Halhy subunit consists of two α/β domains: a small domain (residues 1–114) and a large domain (residues 128–281), connected by an interdomain loop (residues 115–127) (Figure 2A). The closest structural homologs of the Halhy subunit are subunits of BCATs (Table S1). Superposition of the subunits of Halhy and canonical bsDAAT showed less similarity with the corresponding RMSD between Cα of about 1.6 Å. 

The contact analysis showed that Halhy is a homodimer in the crystal. Its functional dimer is organized similarly to the dimers of TAs of PLP fold type IV (Figure 2B). The major differences between dimers of Halhy and canonical bsDAAT are observed in the looped regions (especially in the interdomain loop) and in the region 146–161 (Halhy numbering), which corresponds to the α-helix of the large domain.

### 2.6. The Active Site of Halhy

The residues from both subunits form two symmetrical active sites in the dimer, with the PLP molecule covalently bound via a Schiff base to the catalytic K143. Additionally, PLP is coordinated by the conserved among TAs of PLP fold type IV residues: E176, forming a hydrogen bond with N1 atom of pyridine ring and R52, I202, T203, and T239, forming hydrogen bonds with the phosphate moiety of PLP. The phenyl oxygen atom of PLP forms a hydrogen bond with Y147 located on the α-helix of the large domain. Noteworthy, this hydrogen bond is a hallmark of BCATs. In contrast, in bsDAAT as well as in DAATs from *Burkholderia thailandensis* (PDB ID: 4TM5) and *Burkholderia cenocepacia* (PDB ID: 4PBC), the phenyl moiety is coordinated through water molecule by a tyrosine residue (Y31 in bsDAAT) located on βX-strand of the small domain. However, in Halhy, this position in the βX-strand is occupied by F33, as in canonical BCATs.

**Figure 3:**
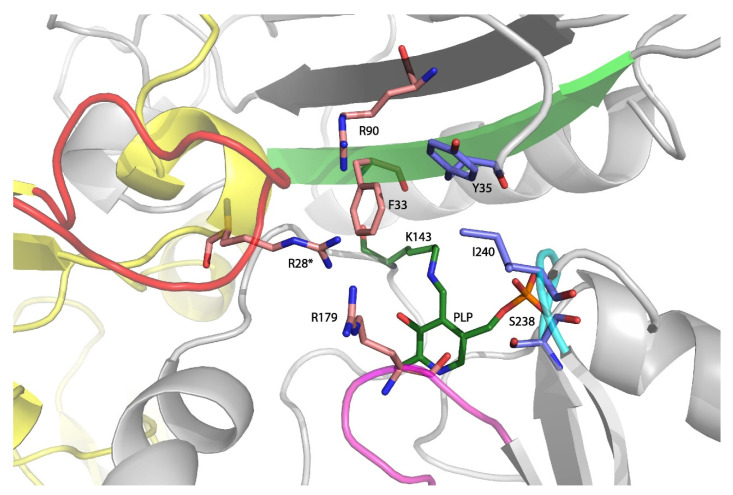
The active site of Halhy. The residues forming the O-pocket and the P-pocket are colored pink and blue, respectively; the PLP molecule and the catalytic lysine are colored dark green. The adjacent subunit is depicted in yellow. The βX-stand and the βY-strand are colored green and black, respectively. The O-pocket loop is colored red. The β-turn ^238^STIK^241^ and the β-turn ^177^SARS^180^ are colored cyan and magenta, respectively. The rest of the molecule is in grey.

Similar to the known TAs of PLP fold type IV, the active site of Halhy can be divided into two pockets: the O-pocket (near the phenyl moiety of PLP) and the P-pocket (near the phosphate moiety of PLP) (Figure 3) [4,25]. According to the enantioselectivity of Halhy, the α-carboxylic group of substrates must be coordinated in the O-pocket, while their side groups must be coordinated in the P-pocket. The O-pocket of Halhy is confined by the side chains of residues F33, R28*, R90, and R179, forming a positively charged patch. The less positively charged O-pocket of bsDAAT is restricted by the side chains of residues Y31, R98*, H100*, L149, and S180. The O-pocket loop of Halhy (^96^GYSPDGYTPVN^106^) is shorter than that of bsDAAT (^94^GTSPRAHQFPENTVK^108^) and does not include typical “carboxylate trap” residues—R98* and H100* (bsDAAT numbering). Instead, in Halhy, the side chains of R28* and R90 are directed into the “carboxylate trap” place and could bind the α-carboxylic group of substrates. In bsDAAT, P-pocket is confined by V33, S240, T242, and K35, which is suggested to coordinate the γ-carboxylic group of α-ketoglutarate [26]. The P-pocket of Halhy is lined by Y35, S238, and I240. Therefore, the P-pocket of Halhy is smaller than the P-pocket of bsDAAT. There are no positively charged residues in the P-pocket of Halhy, and the γ-carboxylic group can be coordinated by the side chain of S238 and the backbone nitrogen atoms of the β-turn ^238^STIK^241^. Interestingly, the amino acid composition of the β-turn of Halhy differs from that of the canonical DAATs and BCATs (^240^STTS^243^ in bsDAAT and ^256^GTAA^259^ in BCAT from *E. coli*). The interdomain loop in the crystal structure of Halhy (^115^DLPASAWEFSAQG^127^) is out of the active site, similarly to other DAATs (PDB ID: 1DAA, 4TM5, and 4PBC).

To summarize, the active site of Halhy differs significantly from that of bsDAAT: the P-pocket is smaller and does not have positively charged residues, the O-pocket does not have a “carboxylate trap,” but instead has three positively charged residues (R28*, R90, and R179) possibly carrying a “carboxylate trap” function. The structural alignment of 27 homologous TAs (Appendix A) showed that four of them have positively charged residues in the R28* position, 19 TAs—in the R90 position, and only Halhy and uncharacterized TA (PDB ID: 6Q1R) have arginine residue in the R179 position in the β-turn ^177^SARS^180^, which is located above the PLP pyridine ring and confines the entrance into the active site through stacking interactions with Tyr102*. To further investigate the role of the unique R179, we constructed the R179G variant of Halhy: glycine was chosen as the most frequently occurred residue in this position (51%) among comparing TAs (Appendix A).

### 2.7. Characterization of the R179G Variant

**Figure 4:**
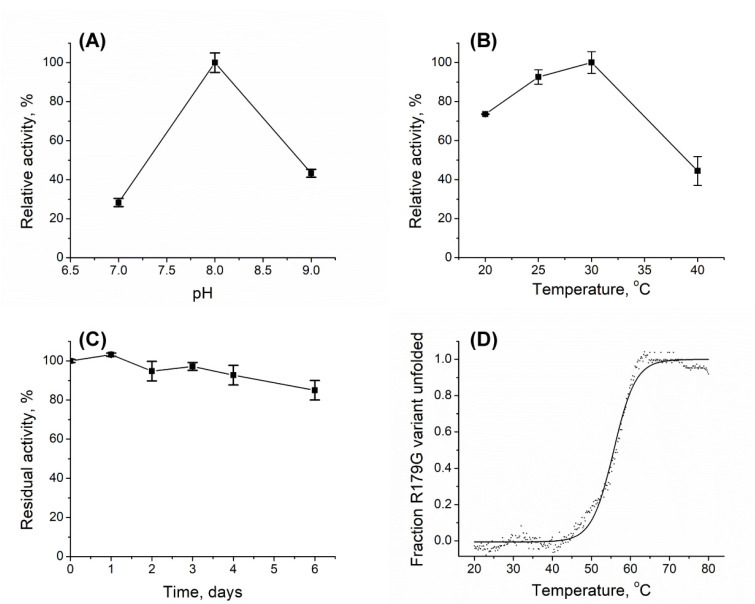
Kinetic and thermodynamic stability of the R179G variant. (**A**,**B**) The effects of pH and temperature on the overall reaction between D-alanine and α-ketoglutarate; 100% corresponds to 0.20 ± 0.01 U/mg (**A**) and 0.32 ± 0.02 U/mg (**B**). (**C**) The residual activity of the R179G variant incubating at 40 °C, 100% corresponds to 0.32 ± 0.02 U/mg in the standard assay. Error bars represent standard deviation. (**D**) Thermal unfolding in K-phosphate buffer, pH 8.0.

The R179G variant was expressed in a soluble form in *E. coli* and purified to homogeneity. The R179G variant eluted similarly to the WT Halhy (Figure S2). The kinetic and thermodynamic stability data of R179G are given in Figure 4. In the standard reaction, the R179G variant displayed the same pH optimum as WT (Figure 4A); however, the temperature optimum decreased to 30 °C (Figure 4B). At the same time, the R179G variant demonstrated stability at 40 °C, pH 8.0 (Figure 4C); the thermal unfolding experiment showed T_0.5_ = 55.7 ± 0.1 °C, which is nearly the same as for WT (Figure 4D). The obtained stability data indicated the structural integrity of Halhy with the R179G substitution and some disturbances in the active site. According to the kinetic parameters of the overall transamination reaction given in Table 4, the R179G variant appeared to be much less active than WT in the optimal conditions. The obtained characteristics support the functional role of R179.

### 2.8. D-Alanine Binding in the Active Site of the WT Halhy and Its R179G Variant

**Figure 5:**
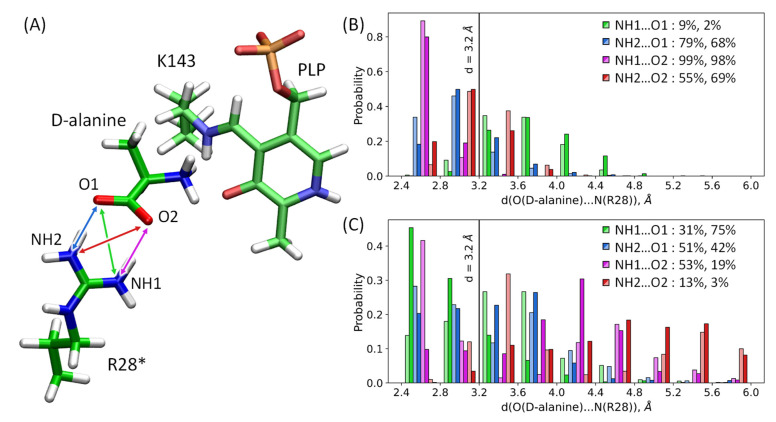
(**A**) A part of the Halhy active site composed of the protonated Schiff base formed by K143 and PLP and R28* with the bound substrate, D-alanine. The hydrogen bond distances measured between nitrogen and oxygen atoms are colored following the histograms. Probability distributions of the hydrogen bond distance in the WT Halhy (**B**) and the R179G variant (**C**). Darker and lighter bars of the same color correspond to the different subunits of the functional dimer. Values in percentages show the fractions of MD trajectories when the hydrogen bonds are formed (the criterion is the distance between the non-hydrogen atoms less than 3.2 Å). The values for the subunits are given separately. Here and in Figure 6, the color code for molecular models is the following: carbon—green, oxygen—red, nitrogen—blue, hydrogen—white, and phosphorus—orange.

**Figure 6:**
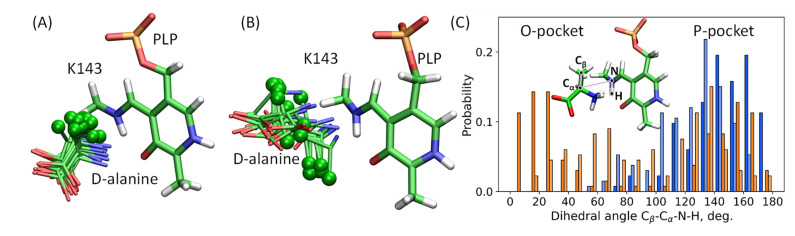
Alignment of multiple frames from the MD runs of the ES complex of WT Halhy (**A**) and the R179G variant (**B**) with D-alanine. (**C**) Distributions of dihedral angles illustrating the orientation of D-alanine. Smaller values correspond to the side chain binding to the O-pocket, larger values—to the P-pocket. Blue bars are for the WT Halhy and orange for the R179G variant. Darker and lighter bars of the same color correspond to the different subunits.

According to the steady-state kinetic experiments (Table 4), the R179G substitution led to a 17-fold decrease in *k_cat_* and a 14-fold increase in *K_m_* of D-alanine. These changes might indicate worse binding of the substrate in the active site. We analyzed MD trajectories of the enzyme-substrate complexes (ES complexes) of the WT Halhy or the R179G variant with D-alanine to quantify these changes. We found that hydrogen bonds between the α-carboxylic group of D-alanine and the side chain of R28* were formed in both model systems (Figure 5). Therefore, we suppose that R28* is responsible for the proper D-alanine binding in the ES complexes. We observed that R90 formed a hydrogen bond with the α-carboxylic group around half of the simulation time, and R179 did not interact with D-alanine during the simulations. Generally, hydrogen bonds between D-alanine and R28* are more stable in the WT Halhy. One hydrogen bond is observed during all simulations (>98% for both subunits), and two hydrogen bonds with the NH2 group exist for longer than half of the simulation time. In the R179G variant, no hydrogen bonds exist during the whole simulation, resulting in the increased flexibility of the substrate. In the active site of WT Halhy, the side chain of D-alanine is located in the P-pocket (see C_β_ atoms in Figure 6A,C), which is in line with the previous studies [3,27]. The P-pocket location corresponds to the dihedral angle C_β_-C-N-H values between 100 and 180 degrees. In contrast, the side chain of D-alanine is highly flexible in the ES complex of the R179G variant (Figure 6B,C) and occupies both P- and O-pockets during the simulations. These findings explain the decrease in the catalytic activity of the R179G variant and worse binding in the active site. 

## 3. Discussion

The PLP-dependent transaminase from *H. hydrossis*, Halhy, is a strict DAAT, active towards D-glutamate, aliphatic and aromatic D-amino acids, and corresponding keto acids. Halhy is distinguished by the high conversion rate of α-ketoglutarate and D-glutamate under reaction conditions typical of known DAATs: pH 8.0–8.5 at 30–40 °C. The functional unit of Halhy is a dimer similar to the dimers of canonical TAs of PLP fold type IV. The Halhy active site comprises several features of canonical BCATs and DAATs. The hydrogen bond betweenY147 and phenyl oxygen of PLP, R90 in βY-strand in the O-pocket and lack of charges in the P-pocket are typical of BCATs, while a loose interdomain loop and a positively charged patch for α-carboxylic group binding in the O-pocket are typical of DAATs. However, the Halhy active site differs significantly in the composition of amino acids from known BCATs and DAATs. A new approach for the proD-coordination of substrates can be observed in Halhy: (1) two arginine residues (R28* and R90) can bind an α-carboxylic group of a substrate; (2) a unique R179 participates in the substrate binding and can control the active site entrance; (3) no positive charges in the P-pocket; however, the residues from β-turn ^238^STIK^241^ can orient properly γ-carboxylic group of D-glutamate/α-ketoglutarate (the side chain of K241 is out of the P-pocket). Finally, residues from the O-pocket loop and the interdomain loop seem not to participate in substrate binding. Earlier, a tandem of arginine and lysine residues in the O-pocket instead of “carboxylate trap” was observed in the active site of the TA from *C. pusillum* (PDB ID: 5K3W) [7], which catalyzed the transamination between D-amino acids or (*R*)-amines and α-keto acids. Another common feature of the active sites of Halhy and TA from *C. pusillum* is an unshaped P-pocket with the interdomain loop aside of it and positively charged residue on the β-turn (^238^STIK^241^ and ^270^SSVR^273^, respectively). However, the O-pocket loop (its amino acid composition) and the active site entrance of TA from *C. pusillum* differ significantly from that of Halhy and canonical BCATs. 

In summary, Halhy is a “high quality” DAAT, and its activity and specificity are achieved at the active site of a unique amino acid composition. We suppose that the DAAT-like specificity among TAs of PLP fold type IV can be achieved at active sites of various amino acid compositions, but having at least two important structural features. These are a strong positively charged patch in the O-pocket (a “carboxylate trap” of various amino acid compositions) and the unshaped P-pocket with an unconserved coordination site for the γ-carboxylic group of α-ketoglutarate

## 4. Materials and Methods

### 4.1. Cloning, Expression, and Purification of the Recombinant Halhy and Its R179G Variant

The gene Halhy_2446 encoding transaminase of PLP fold type IV was identified in the complete genome sequence of *H. hydrossis* using TA with an expanded substrate specificity from *C. pusillum* [7] as a query sequence. The nucleotide sequence of Halhy_2446 was optimized for expression in *E. coli* using server Optimizer (http://genomes.urv.es/OPTIMIZER/) and synthesized by ATG Service Gene (St. Petersburg, Russia) with overhangs complementing the NdeI and HindIII restriction sites attached to the 5′ and 3′-ends, respectively. The synthetic gene was cloned into the pET-21d vector (Novagen, Darmstadt, Germany) modified as described by Boyko et al. [28]. Recombinant Halhy (281 a.a., 32.09 kDa) fused at the N-terminus to a His6TEV-tag was expressed in *E. coli* Rosetta(DE3) pLysS cells (Novagen, Darmstadt, Germany). Transformed cells were grown in LB high salt medium, containing 100 µg/ml ampicillin and 34 µg/ml chloramphenicol (Panreac-AppliChem, Darmstadt, Germany) at 37 °C until the OD600 value reached 0.8, and the expression was induced with 0.2 mM IPTG. After incubation for 18 h at 25 °C, the cells were harvested by centrifugation, resuspended in 50 mM Tris-HCl buffer, pH 8.0, supplemented with 500 mM NaCl, 20 mM imidazole, 1 mM b-mercaptoethanol, 0.1% (*v*/*v*) Triton X-100, 100 µM PLP, and 1 mM PMSF, and disrupted by sonication. The crude cell extract was centrifuged for 25 min at 18,500 × g. The supernatant was applied to a HisTrap HP column (Cytiva, Marlborough, MA, USA), equilibrated in a binding buffer (50 mM Tris-HCl buffer, pH 8.0, supplemented with 500 mM NaCl, 20 mM imidazole, and 0.1% (*v*/*v*) Triton X-100). The (His)6-tagged recombinant Halhy was eluted with a linear gradient from 20 to 500 mM imidazole in the same buffer without Triton X-100. The active pool was incubated for 1 h with a ten-fold molar excess of PLP at +4 °C, then concentrated with a 30 kDa cut-off centrifugal filter device (Millipore, Burlington, MA, USA) and transferred into 50 mM Tris-HCl buffer, pH 8.0, supplemented with 100 mM NaCl, 1 mM EDTA, 1 mM β-mercaptoethanol, 5% (*v*/*v*) glycerol, and TEV protease (1 mg per 10 mg of the protein). The solution was incubated for 2 h at room temperature, dialyzed against the binding buffer, and applied to a HisTrap HP column. TEV protease and a cleaved His-tag were absorbed on the column, and the flow-through was concentrated and applied to a Superdex 200 10/300 GL column (Cytiva, Marlborough, MA, USA) equilibrated in 50 mM HEPES buffer, pH 7.5, supplemented with 100 mM NaCl, 1 mM DTT, and 100 µM PLP. Active fractions of Halhy were stored at −20 °C with glycerol added to a final concentration of 50%. The protein purity was analyzed by SDS-PAGE (12%). The protein concentration was determined spectrophotometrically [29].

The R179G variant of Halhy was created by easy single-primer site-directed mutagenesis as described in [30,31]. Eighteen cycles of PCR amplification were performed on an expression plasmid carrying Halhy_2446 gene using Tersus Plus PCR kit (Evrogen, Moscow, Russia) and a mutagenesis primer 5′-AGGAAGAAGTTAGAGCCAGCGCTTTCACGAATCC-3′, the mutation is underlined. Amplified fragments were treated with the DpnI (Thermo Fisher Scientific, Waltham, MA, USA) restriction enzyme to eliminate the methylated DNA template, and were then transformed to *E. coli* Mach I cells. Clones carrying target mutation were identified by colony PCR assay performed using Taq DNA polymerase and the pair of primers consisting of T7 universal forward primer and check primer 5′-CAGGAAGAAGTTAGAGCC-3′. To verify the introduced mutation and ensure the absence of random mutations associated with PCR, selected clones were sequenced on an ABI 3730xl DNA Analyzer (Applied Biosystems, Foster City, CA, USA). The R179G variant production and purification were performed as described for wild-type Halhy.

The (*R*)-2-hydroxyglutarate dehydrogenase (HGDG) from *Acidaminococcus fermentans* was produced and purified similarly. The purified enzyme was desalted in 100 mM K-phosphate buffer, pH 8, and stored at −20 °C in 50% glycerol.

The amino acid sequences were checked by MALDI-TOF MS analysis (UltraFlextreme Bruker Daltonik, Bremen, Germany).

### 4.2. Enzyme Activity Assay

The overall transamination reaction with D-amino acids was assayed at 40 °C by coupled enzyme reaction using lactate dehydrogenase (LDH) (Sigma, St. Louis, MO, USA) to detect pyruvate production. Standard assay solution contained 50 mM K-phosphate buffer, pH 8, 30 µM PLP, 5 mM D-alanine and 2 mM α-ketoglutarate, 330 µM NADH, and 2 U/ml LDH. When D-glutamate was taken in the transamination reaction, 4 U/ml HGDH was applied to detect α-ketoglutarate production, the reaction mixture contained 50 mM K-phosphate buffer, pH 8, 30 µM PLP, 5 mM D-glutamate and 2 mM α-keto acid, and 330 µM NADH. The reaction was initiated by adding 0.01–10 µM of the purified Halhy. The reaction progress was monitored by detection the decrease in the absorbance (ε_NADH_ = 6220 M^−^^1^ cm^−^^1^) at 340 nm, using SPECTROstar Omega (BMG Labtech GmbH, Ortenberg, Germany). For measuring the activity at 50 °C and above, and a pH above 8.5, the pyruvate formation was monitored using a discontinuous lactate dehydrogenase assay at 25 °C, in 100 mM K-phosphate buffer, pH 7.0 [32]. The activity of Halhy was calculated from the initial linear region of the progress curve of the reaction. One unit (U) was defined as the amount of the enzyme that catalyzed the conversion of 1 µmol of the substrate into a product per minute. 

Steady-state kinetic parameters of the direct reaction between D-alanine and α-ketoglutarate and the reverse reaction between D-glutamate and pyruvate were determined from the substrate saturation curves at the constant co-substrate concentration at 40 °C. Saturation curves were analyzed using the Michaelis-Menten model. The kinetic parameters were calculated by fitting the initial velocity data to Equation (1):

(1) $$V=\frac{V_{m}\times A\times B}{K_{M}^{A}\times B+K_{M}^{B}\times A+A\times B\;}$$

where V is the initial velocity, $V_{m}$ is the maximum velocity, A and B are substrate concentration, and $K_{M}^{A}$ and $\;K_{M}^{B}$ are the $K_{M}$ of substrates *A* and *B*, respectively. All measurements were performed at least in triplicate. The data were analyzed using Origin 8.0 software (OriginLab, Northampton, MA, USA). The activity of the R179G variant was measured in the same way at 25 °C.

The activity towards (*S*)-(−)- and (*R*)-(+)-1-phenylethylamine (PEA) was measured spectrophotometrically at 40 °C by the acetophenone assay [33], using Evolution 300 UV-Vis spectrophotometer (Thermo Scientific, Waltham, MA, USA). The assay mixture contained 50 mM CHES buffer, pH 9, 30 µM PLP, 5 mM amine, and 2 mM α-ketoglutarate or pyruvate. An extinction coefficient (ε) of 11,600 M^−1^ cm^−1^ was used for acetophenone. One activity unit was defined as the amount of the enzyme necessary to form 1 μM of acetophenone per minute.

The activity towards D-leucine and L-leucine was measured chromatographically by analyzing the production of 4-methyl-2-oxovalerate. The assay mixture contained 50 mM K-phosphate buffer, pH 8, 30 µM PLP, 5 mM D- or L-leucine, and 2 mM α-ketoglutarate at 40 °C. The reaction was initiated by the addition of 1.2 µM Halhy. The aliquots were taken at 10 minute time intervals. The reaction was terminated by heating up to 95 °C for 5 minutes, followed by centrifugation at 10,000× *g* for 10 min. The aliquots were applied to the C18 column (Zorbax Eclipse XDB-C18, 5 µm, 4.6 × 150 mm, (Agilent Technologies, Inc., Santa Clara, CA, USA)). The column was equilibrated in 20 mM KH_2_PO_4_, pH 3, containing 15% methanol, at 1.0 ml/min, 25 °C. The product accumulated during the reaction was eluted with the same retention time (8.6 min) as the control 4-methyl-2-oxovalerate sample. One unit (U) was defined as the amount of the enzyme necessary to form 1 µmol 4-methyl-2-oxovalerate per minute.

### 4.3. Effect of pH and Temperature on the Transamination Reaction

The optimal pH and temperature were determined for the overall transamination reaction between 5 mM D-alanine and 2 mM α-ketoglutarate. The Halhy activity was measured in a mixed buffer containing 50 mM Tris-HCl and 50 mM K-phosphate, pH 6–9, supplemented with 30 µM PLP at 30 °C. The optimal temperature was determined at a temperature range from 15 to 60 °C in 50 mM K-phosphate buffer, pH 8.0, and 30 µM PLP.

### 4.4. Analysis of Halhy Thermal Stability

The thermal stability was determined by incubating 30 µM Halhy at 40 °C in 50 mM K-phosphate buffer, pH 8.0, containing 50 mM NaCl and 100 µM PLP. Residual activity was measured in the standard assay at regular intervals for six days.

The thermal unfolding was studied using circular dichroism spectroscopy. Molar ellipticity [θ] changes at 208 nm were measured for 10 µM Halhy in 50 mM K-phosphate buffer, pH 8, in a 1-mm optical path cuvette using a Chirascan instrument, equipped with a Peltier thermostatic cell holder (Applied Photophysics, Surrey, UK). Heating from 20 to 80 °C was carried with 1 °C/min. The midpoint temperature of thermal denaturation (T_0.5_) was calculated using Boltzmann’s equation.

### 4.5. Analysis of the Product Yield and Enantiomeric Excess in the Transamination Reaction

The product yield was determined by monitoring the consumption of 4-methyl-2-oxovalerate in reaction *4-methyl-2-oxovalerate + D-glutamate* or the production of D-phenylalanine in the reaction *phenylpyruvate + D-glutamate*. A one-pot three-enzyme system was employed to shift the equilibrium of the reaction towards the products. In this process, the coproduct, α-ketoglutarate, was removed from the reaction medium by HGDG assay, accompanied by NADH recovery using glucose dehydrogenase reaction with glucose. The reaction mixture contained 100 mM K-phosphate buffer, pH 7.5, 100 µM PLP, 50 mM D-glutamate, 50 mM 4-methyl-2-oxovalerate or 50 mM phenylpyruvate, 1 mM NADH, 150 mM D-glucose, 90 U HGDH, and 50 U glucose dehydrogenase (Sigma, St. Louis, MO, USA). The reaction mixtures were incubated at 30 °C for 20 h. The reactions were terminated by removing the enzyme using an Amicon-Ultra-15 centrifugal tube (Millipore, Burlington, MA, USA), and then aliquots were analyzed by HPLC (ӒKTA Purifier, Cytiva, Marlborough, MA, USA).

4-Methyl-2-oxovalerate and D-phenylalanine were directly detected by HPLC using a reverse-phase C18 column (Zorbax Eclipse XDB-C18, 5 µm, 4.6 × 150 mm, (Agilent Technologies, Inc., Santa Clara, CA, USA)) with a UV detector set at 210 nm. The column was equilibrated in 20 mM KH_2_PO_4_, pH 3, containing 15% methanol, at 1.0 mL/min, and at 25 °C. Retention times of pure 4-methyl-2-oxovalerate and D-phenylalanine were 8.6 and 4.3 min, respectively. 

The chiral analysis of produced D-leucine and D-phenylalanine was performed by HPLC using a reverse-phase C18 column with a UV detector set at 340 nm. Deproteinized samples were derivatized with Marfey’s reagent (Sigma, St. Louis, MO, USA) [34].

### 4.6. Crystallization and Data Collection

The holo form of the Halhy was crystallized by the “hanging drop” vapor diffusion method in 24-well VDX plates (Hampton Research, Aliso Viejo, CA, USA). A total of 1.5 µl of protein (15 mg/ml) was mixed with 1.5 µL of precipitant containing 0.1 M Sodium acetate buffer, pH 4.8, and 18% PEG3350 and set up over 500 µl of precipitant in a sealed reservoir. Before data collection, crystals were briefly soaked in the mother liquor containing 25% glycerol as a cryoprotectant. The crystals were then flash-cooled to 100 K in liquid nitrogen. The X-ray diffraction data were collected at the ID30A-3 beamline of the ESRF synchrotron (Grenoble, France). The data were indexed, integrated, and scaled using Dials [35]. The program Pointless [36] suggested a C2 space group with one subunit per asymmetric unit. The data collection and processing statistics are summarized in Table 5.

### 4.7. Structure Solution and Refinement

Halhy structure was solved by the molecular replacement method using the Molrep program [37] with the atomic coordinates of the branched-chain aminotransferase from *Geoglobus acetivorans* (PDB ID: 5E25) as a starting model. The refinement of the structure was carried out using the REFMAC5 program of the CCP4 suite [38]. The visual inspection of electron density maps and the manual rebuilding of the model was carried out using the COOT interactive graphics program [39]. A resolution was cut to 2.0 Å during the refinement to reduce the noise of the maps and achieve better R-factors. The refinement statistic is given in Table 5. In the final model, an asymmetric unit contained one independent copy of the protein with 282 visible residues and covalently bound PLP molecule (Supplementary Figure S8), 171 water molecules, one phosphate, and one acetate ion from the buffer and crystallization solution, correspondingly.

The visual inspection of the modeled structures and figure preparation were carried out by the COOT program [39] and the PyMOL Molecular Graphics System, Version 2.4. (Schrödinger, New York, NY, USA). The structure comparison and superposition were made using the PDBeFOLD program [40]. The contacts were analyzed using the PDBePISA [41].

### 4.8. Molecular Modeling

Molecular dynamics simulations were performed using NAMD3 software [42]. A crystal structure obtained in this study was utilized as a source of coordinates of non-hydrogen atoms. Protonation states of amino acid residues were as follows: positively charged R and K, negatively charged E and D, and neutral H. The system was solvated in a rectangular water box so that the distance from the protein surface to the border of the cell exceeded 12 Å. Protein macromolecule was composed of two subunits, and it was described using CHARMM36 force field [43,44] and water molecules with the TIP3P force field parameters [45]. Force field parameters for the PLP molecules were obtained on the web service (https://cgenff.umaryland.edu/) that automatically assigns atom types of the CGenFF to all atoms [46]. We obtained a patch to describe a protonated Schiff base formed by the PLP and the K143 of the transaminase. We compared the dynamic behavior of the WT and R179G variant of Halhy complexed with the D-alanine substrate in the active site (ES complex). To model ES complexes within the classical force field parameters, we added harmonic potentials to the following distances: a distance between the hydrogen atom of the protonated amino group of D-alanine and a nitrogen atom of the protonated Schiff base with the ξ_0_ = 2 Å and K = 40 kcal/mol/Å^2^, a distance between the nitrogen atom of D-alanine and the carbon atom of the protonated Schiff base with the ξ_0_ = 3.2 Å and K = 40 kcal/mol/Å^2^. The NPT ensemble was utilized with *p* = 1 atm and T = 300 K with the 1 fs integration time step; the total length of the MD trajectory for each model system was 200 ns. Cutoff distances for all non-covalent interactions were 12 Å with the switch to the smoothing potential at 10 Å.

## 5. Conclusions

We described the D-amino acid transaminase from bacteria *H. hydrossis*: the new enzyme is strictly specific towards D-amino acids and α-keto acids and *in vitro* catalyzes the transamination between D-glutamate and pyruvate with one of the highest rate constants among DAATs. Furthermore, Halhy is attractive as a potential biocatalyst because of its stability at 40–50 °C. We succeeded in obtaining the crystal structure of the holo form of Halhy with a resolution of 2.0 Å. The structural analysis revealed that the scaffold of the Halhy subunit is typical of TAs of PLP fold type IV. However, the combination of residues, determining the substrate specificity, is new: three arginine residues (R28*, R179, and R90) were revealed in the O-pocket. We performed molecular dynamic simulations and demonstrated that the residue R28* is responsible for D-amino acid-binding in the active site due to stable hydrogen bonds with the carboxylic group. R90 appears to assist R28* in substrate binding. We created the R179G variant of Halhy and found that the R179G mutation led to the worsening of the catalytic properties of Halhy (both *k_ca_*_t_ and *K_m_*); molecular dynamic simulations revealed that this is due to the decrease in the hydrogen bonds stability between the substrate and R28* manifested in the increased flexibility of the substrate. The observed *sequence-structure-function* relationships in DAATs and the members of the superfamily of TAs of PLP fold type IV in whole show the existence of various ways to achieve the desired specificity within the TA scaffold. However, changing the specificity of TAs is still a complex task in bioengineering.

## Figures and Tables

**Table 1:** The specificity-determining sequence motifs of the canonical DAATs, BCATs, R-TAs, TA with an expanded substrate specificity from *C. pusillum* and TA from *H. hydrossis* (Halhy), according to Höhne et al. [6].

TA, (Numbering Corresponds to)	Specificity-Determining Sequence Motifs	
	Motif 1	Motif 2
DAAT, (DAAT from *Bacillus* sp. YM-1)	^26^FxxxxYxV[IVA][KR]^35^	^86^HxY^88^…^98^[RK]xH^100^
BCAT, (BCAT from *Escherichia coli*)	^31^YxxxxF[ED]Gx[KR]^40^	^95^YxR^97^…^107^[LMVI]G[VL]^109^
R-TA, (R-TA from *Aspergillus terrus*)	^55^HxxxxYD[VT]x[STAHP]^64^	^115^[FY]V[EQAWNS]^117^… ^128^[RKFGP]x[STANER]^130^
TA from *C. pusillum*	^51^RxxxxFETIA^60^	^115^GxK^117^…^123^GIEGEGR^129^
Halhy	^28^RxxxxFDYFL^57^	^88^GxR^90^…^96^GYSPDGYTPVN^106^

**Table 2:** The activity of Halhy towards different amino donors in the overall transamination reaction.

Amino Donor	Amino Acceptor	Initial Activity, U/mg	Assay
D-alanine	α-ketoglutarate	50 ± 3	LDH
D-leucine		0.70 ± 0.08	HPLC
L-alanine		ND	LDH
L-leucine		ND	HPLC
(*S*)-PEA		ND	Acetophenone assay
(*R*)-PEA		ND	Acetophenone assay
D-glutamate	pyruvate	74 ± 2	HGDH
(*S*)-PEA		ND	Acetophenone assay
(*R*)-PEA		ND	Acetophenone assay

ND—not detected.

**Table 3:** The activity of Halhy in the overall reaction with D-glutamate and various α-keto acids.

α-Keto Acid	Initial Activity, U/mg
pyruvate	74 ± 2
α-ketoglutarate	50 ± 3 (with 5 mM D-alanine)
2-oxobutyrate	42 ± 2
2-oxovalerate	15.0 ± 0.4
2-oxohexanoate	0.320 ± 0.005
3-metyl-2-oxovalerate	0.085 ± 0.004
4-metyl-2-oxovalerate	0.220 ± 0.007
phenylpyruvate	1.88 ± 0.02

**Table 4:** The steady-state kinetic parameters of the overall transamination reaction catalyzed by wild-type Halhy (WT) and its R179G variant.

Substrate	Cosubstrate	*k_cat_*, c^−1^	*K_m_*, mM	*k_cat_/K_m_*, c^−1^ M ^−1^
*Wild-type Halhy*				
α-ketoglutarate	D-alanine 40 mM	146 ± 4	2.3 ± 0.2	63,000 ± 7000
D-alanine	α-ketoglutarate 5 mM	146 ± 4	23 ± 1	6300 ± 400
pyruvate	D-glutamate 4 mM	215 ± 6	2.1 ± 0.1	103,000 ± 8000
D-glutamate	pyruvate 2.5 mM	215 ± 6	10.3 ± 0.7	21,000 ± 2000
D-leucine	α-ketoglutarate 5 mM	10.5 ± 0.4	83 ± 8	130 ± 20
4-methyl-2-oxovalerate	D-alanine 40 mM	4.2 ± 0.1	70 ± 6	60 ± 7
*R179G variant*				
α-ketoglutarate	D-alanine 40 mM	8.6 ± 0.6	8.1 ± 0.8	1100 ± 200
D-alanine	α-ketoglutarate 5 mM	8.6 ± 0.6	321 ± 32	27 ± 5

**Table 5:** Data collection, processing, and refinement.

Diffraction Source	ID30A-3, ESRF
Wavelength (Å)	0.97
Temperature (K)	100
Detector	Eiger X 4M
Crystal-to-detector distance (mm)	131.78
Rotation range per image (°)	0.2
Total rotation range (°)	160
Space group	C2
*a*, *b*, *c* (Å)	86.88, 71.75, 52.99
α, β, γ (°)	90.0, 100.96, 90.0
Resolution range (Å)	52.03–2.00 (2.04-2.00)
Number of unique reflections	21,396 (1419)
Completeness (%)	97.4 (92.5)
Average redundancy	3.0 (3.2)
〈*I*/σ(*I*)〉	5.2 (1.9)
Rmeas (%) (Diederichs and Karplus 1997)	13.4 (57.6)
CC_1/2_ (Diederichs and Karplus 1997)	98.7 (87.1)
Number of reflections used for the refinement	20,186
Rfact (%)	18.0
*R*_free_ (%)	24.4
Bonds (Å)	0.02
Angles (°)	1.95
Average B-factors (Å^2^)	
Protein	27.9
Water	30.5
PLP	25.5
Ramachandran plot	
Most favoured (%)	92.5
Allowed (%)	5.7
PDB entry code	7P7X

**Figure A1:**
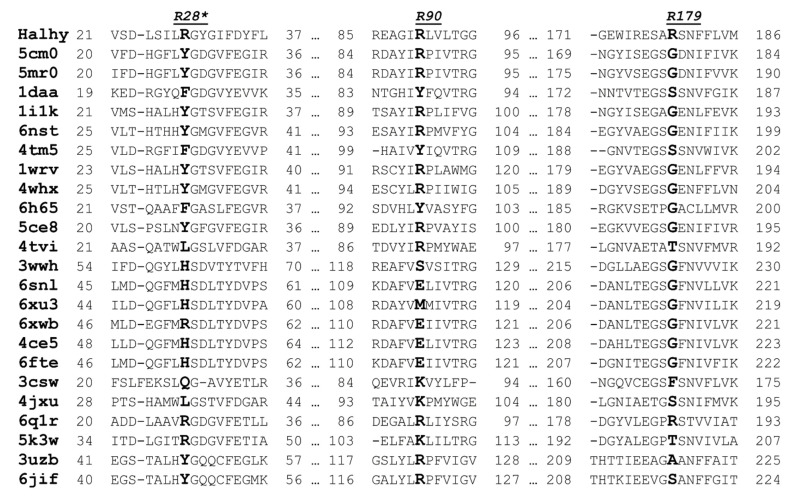
The multiple structure alignment of Halhy and its close structural homologs (RMSD between Cα 1.4-2.0 Å) referred to by the PDB codes. The amino acid residues in the equivalent positions of R28*, R90, and R179 (numbering corresponds to Halhy) are marked in bold.

## Data Availability

The data presented in this study are available on request from the corresponding author.

## Supplementary Materials

Table S1: Superposition of the Halhy subunit with the homologous TA of PLP fold type IV; Figure S2: The gel filtration elution profile for Halhy; Figure S3: The absorption spectrum of Halhy; Figure S4: pH-dependence of the Halhy spectrum in the PLP form; Figure S5: HPLC analysis of the configuration of the products of the overall transamination reaction catalyzed by Halhy; Table S2: HPLC analysis condition; Table S3: Retention times of isomers of leucine and phenylalanine after derivatization with Marfey’s reagent. Figure S6: Steady-state kinetics of the transamination reactions catalyzed by Halhy; Figure S7: Steady-state kinetics of the transamination reaction catalyzed by the R179G variant. Figure S8: Omit density map for the PLP molecule in Halhy holo structure.

## Sample Availability

Samples of the compounds are available from the authors.
