# Supplementary material for: The Uncommon Active Site of D-Amino Acid Transaminase from Haliscomenobacter hydrossis: Biochemical and Structural Insights into the New Enzyme
Source: Molecules. 2021 Aug 20;26(16):5053. doi: 10.3390/molecules26165053 (PMC8401098; doi:10.3390/molecules26165053)
Supplement: Supplementary file 1 [file molecules-26-05053-s001.zip › molecules-1331229-supplementary.pdf]

molecules

The Uncommon Active Site of D-amino Acid Transaminase from Haliscomenobacter hydrossis: Biochemical and Structural Insights into the New Enzyme

Alina K. Bakunova [1], Alena Yu. Nikolaeva [1], Tatiana V. Rakitina [1,2], Tatiana Y. Isaikina [1], Maria G. Khrenova [1,3], Konstantin M. Boyko [1], Vladimir O. Popov [1] and Ekaterina Yu. Bezsudnova [1,*]

[1] Bach Institute of Biochemistry, Research Center of Biotechnology of the Russian Academy of Sciences, Leninsky Ave. 33, bld. 2, 119071 Moscow, Russian Federation; a.bakunova@fbras.ru (A.K.B.); aishome@mail.ru (A.Y.N.); inbimaldi@gmail.com (T.Y.I.); boiko_konstantin@inbi.ras.ru (K.M.B.); vpopov@inbi.ras.ru (V.O.P.)

[2] Shemyakin&Ovchinnikov Institute of Bioorganic Chemistry of the Russian Academy of Sciences , Miklukho-Maklaya str. 16/10, 117997 Moscow, Russian Federation; taniarakitina@yahoo.com

[3] Chemistry Department, Lomonosov Moscow State University, Leninskiye Gory 1/3, Moscow, 119991, Russian Federation; mkhrenova@lcc.chem.msu.ru

* Correspondence: author E-mail: eubez@inbi.ras.ru

Molecules Supplementary

Table S1. Superposition of the Halhy subunit with the homologous TA of PLP fold type IV.

TA from (PDB ID)	Type	RMSD, Å	Z-Score	Sequence Identity, %	Percentage of the Aligned Residues, %
Geoglobus acetivorans (5CM0)	BCAT	1.4	16.8	29.8	95
Escherichia coli (1I1K)	BCAT	1.4	16.1	24.6	95
Archaeoglobus fulgidus (5MR0)	BCAT	1.4	16.5	29.6	95
Pseudomonas aeruginosa (6NST)	BCAT	1.6	15.2	28.1	95
Aspergillus terrus (4CE5)	R-TA	1.6	14.8	23.7	100
Exophiala xenobiotica (6FTE)	R-TA	1.6	13.4	25.5	84
Bacillus sp.(strain YM-1) (1DAA)	DAAT	1.6	15.8	25.9	89
Burkholderia thailandensis (4TM5)	DAAT	1.7	15.2	25.3	95
*Arthrobacter sp.*KNK*16* (3WWH)	R-TA	1.7	13.7	24.7	95
Haliangium ochraceum (6H65)	TA*	1.7	14.1	26.9	95
Curtobacterium pusillum (5K3W)	TA*	2.0	13.6	29.8	95

*—TA with an expanded substrate specificity.

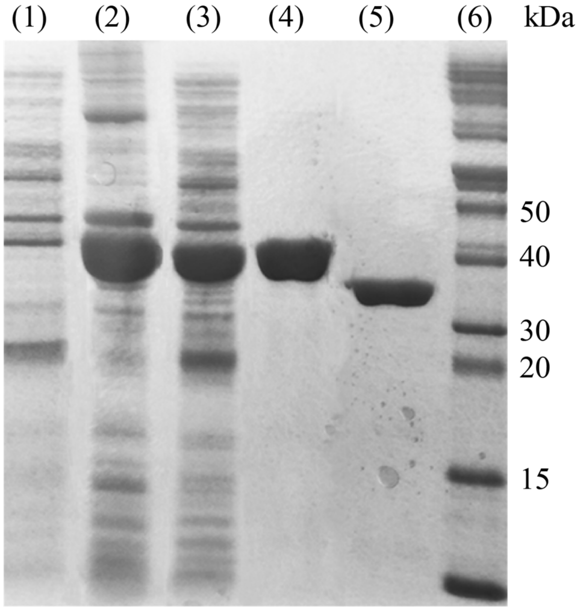

Figure S1. SDS-PAGE of stage expression and purification of Halhy. (**1**) *E. coli* cell before induction; (**2**) *E. coli* cell after IPTG induction; (**3**) cell free lysate obtained by sonication and centrifugation; (**4)** fraction Halhy after HisTrap HP column (Cytiva, Marlborough, MA, USA); (**5**) fraction Halhy after cleavage His-tag, using TEV-protease and gel filtration chromatography; (**6**) Page Ruler Unstained Protein Ladder (Thermo Fisher Scientific, Waltham, MA, USA).

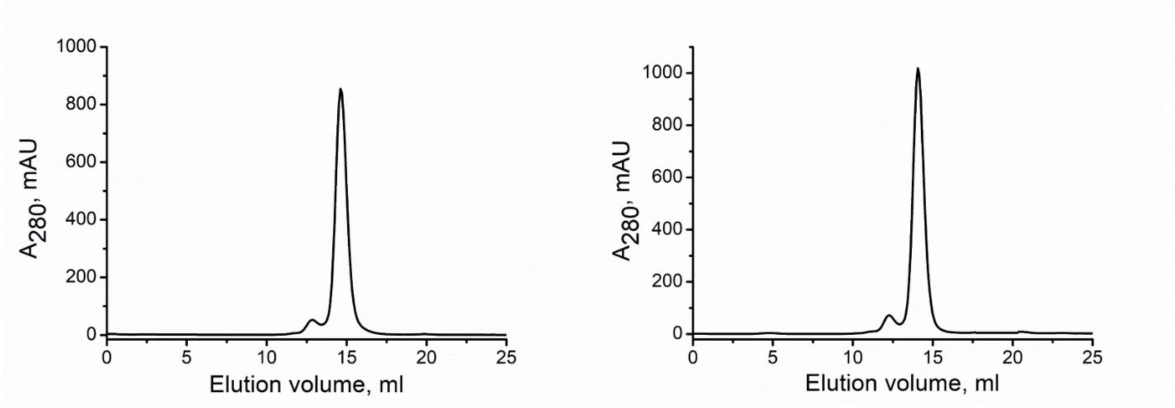

Figure S2. The gel filtration elution profile for Halhy. The major peak corresponds to a dimer of the wild-type Halhy (left) and the variant R179G (right). Column size and specification—A calibrated Superdex 200 10/300 GL column (Cytiva, Marlborough, MA, USA), bed volume 24 ml is equilibrated with 50 mM HEPES buffer, pH 8.0, containing 100 mM NaCl, 100 μM PLP.

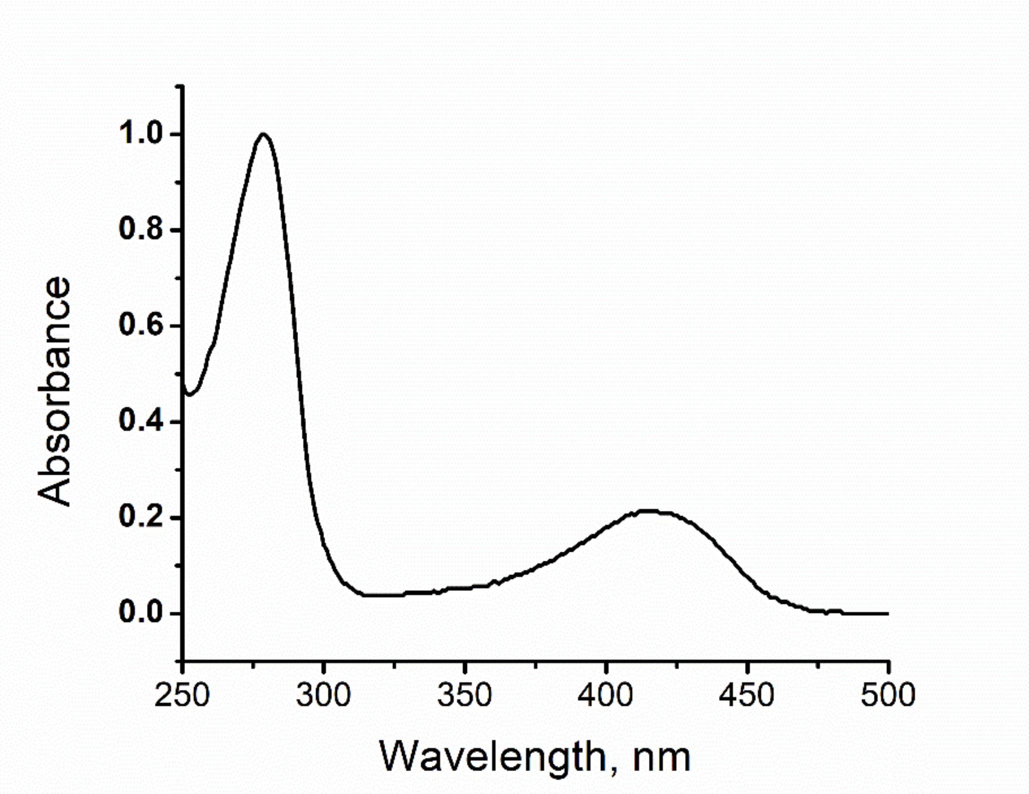

Figure S3. The absorption spectrum of Halhy. Condition: 30 μM PLP form of Halhy in 50 mM K-phosphate buffer, pH 8, at 25 °C.

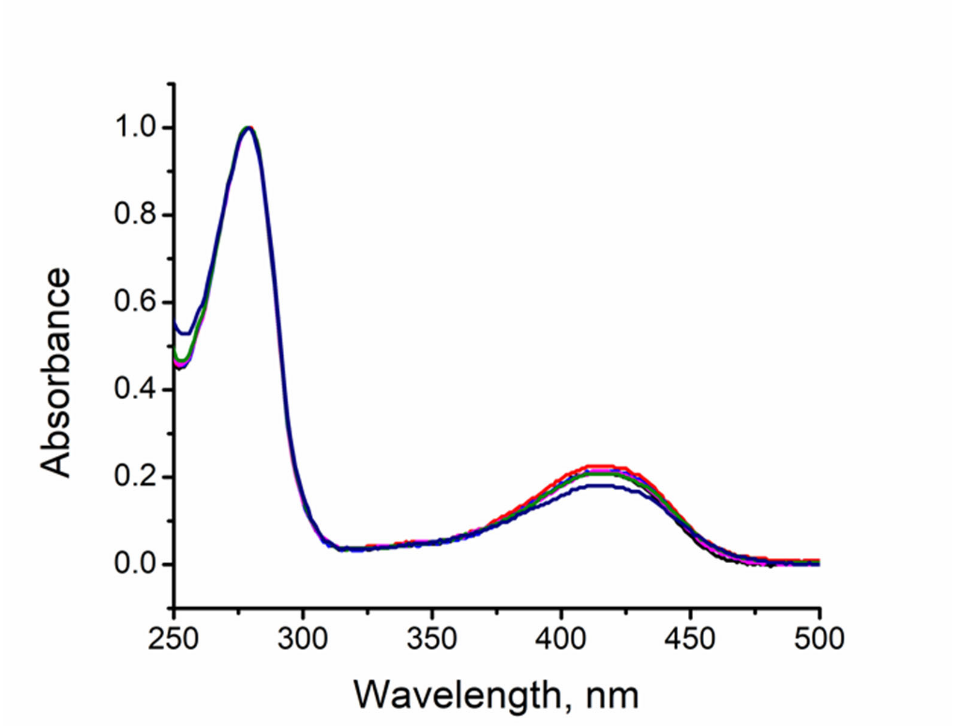

Figure S4. pH-dependence of the Halhy spectrum in the PLP form. The absorption spectrum of 30 μM Halhy in the PLP form in different buffers: 50 mM Na-acetate buffer, pH 4; 50 mM K-phosphate buffer, pH 6–8; 50 mM CHES buffer, pH 9-10, at 25 °C. PLP form was obtained by incubating fractions of Halhy with a ten-fold molar excess of PLP and α-ketoglutarate overnight at 4 °C and further desalting using an appropriate buffer. The absorption spectra were collected using the spectrophotometer Evolution 300 (Thermo Scientific, Waltham, MA, USA).

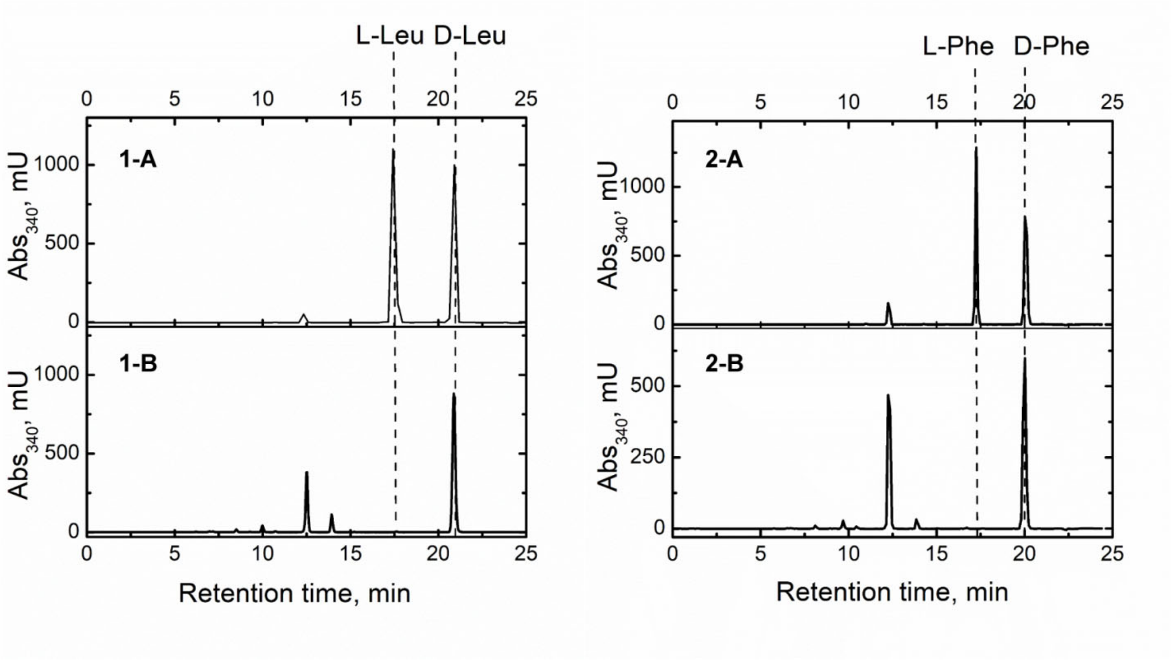

Figure S5. HPLC analysis of the configuration of the products of the overall transamination reaction catalyzed by Halhy. Chromatograms of standards and samples (leucine and phenylalanine) derivatized with Marfey's reagent. **1-A**: reference standards of D- and L-leucine at concentration of 50 mM; **1-B**: the reaction I sample. **2-A**: reference standards of D- and L-phenylalanine at a concentration of 50 mM; **2-B**: the reaction II sample.

Reaction I: 4-methyl-2-oxovalerate + D-glutamate ↔ D-leucine + α-ketoglutarate

Reaction II: phenylpyruvate + D-glutamate ↔ D-phenylalanine + α-ketoglutarate

The chiral analysis of products of reactions I and II—D-leucine and D-phenylalanine—was performed by HPLC using the reverse-phase C18 column with the UV detector set at 340 nm. Deproteinized samples were derivatized with Marfey's reagent (Sigma, St. Louis, MO, USA) according to Bae et al [1]. Briefly, 25 µl of Marfey's reagent (28 mM in acetonitrile) and 10 µl 1 M $NaHCO_3$ were added to 10 µl sample and incubated at 50 °C for 2 h. The reaction mixture was cooled, and then the reaction was stopped by adding 3 µl of 4 M HCl and 10 µl of 100% methanol.

Table S2. HPLC analysis condition.

**Instrument:**	**ÄKTA Purifier, Cytiva, Marlborough, MA, USA**
**Column:**	Zorbax Eclipse XDB-C18, 5 µM, 4.6 × 150 mm, Agilent Technologies, Inc., Santa Clara, CA, USA
**Buffer A:**	0.1% trifluoroacetic acid in water
**Buffer B:**	0.1% trifluoroacetic acid in 100% methanol
**Elution:**	linear gradient of Buffer B from 20% to 70% in 15 min.
**Flow rate:**	1.0 ml/min
**Temperature:**	25 °C
**Injection volume:**	10 µL
**Detection:**	UV, 340 nm

Table S3. Retention times of isomers of leucine and phenylalanine after derivatization with Marfey's reagent.

**Compound**	**RT, min**	
	**L-isomer**	**D-isomer**
Leucine	17.4	20.8
Phenylalanine	17.1	20.0

Reaction I: 4-methyl-2-oxovalerate + D-glutamate ↔ D-leucine + α-ketoglutarate

Reaction II: phenylpyruvate + D-glutamate ↔ D-phenylalanine + α-ketoglutarate

The chiral analysis of products of reactions I and II—D-leucine and D-phenylalanine—was performed by HPLC using the reverse-phase C18 column with the UV detector set at 340 nm. Deproteinized samples were derivatized with Marfey's reagent (Sigma, St. Louis, MO, USA) according to Bae et al [1]. Briefly, 25 µl of Marfey's reagent (28 mM in acetonitrile) and 10 µl 1 M $NaHCO_3$ were added to 10 µl sample and incubated at 50 °C for 2 h. The reaction mixture was cooled, and then the reaction was stopped by adding 3 µl of 4 M HCl and 10 µl of 100% methanol.

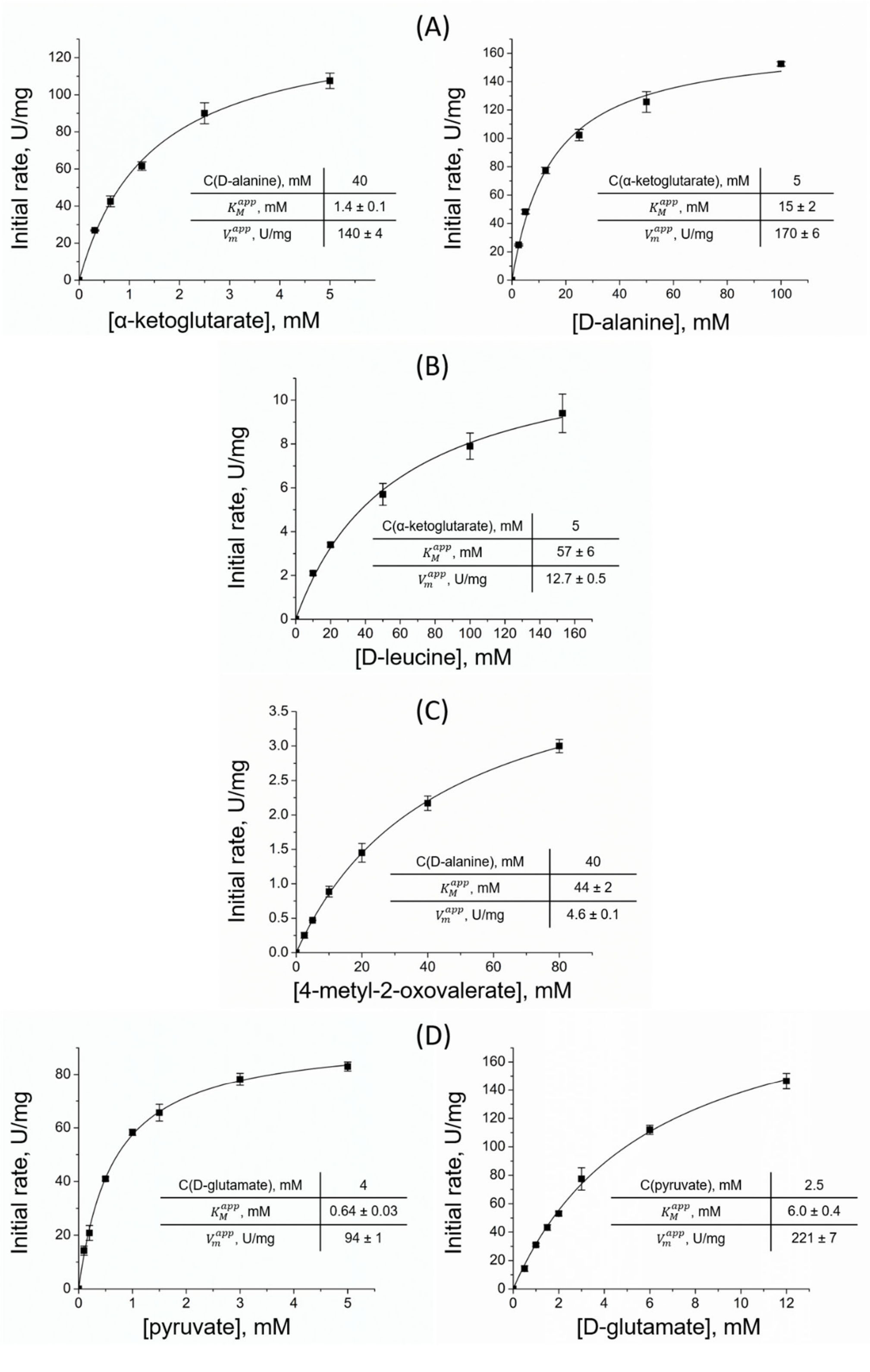

Figure S6. Steady-state kinetics of the transamination reactions catalyzed by Halhy. Reactions: (A) α-ketoglutarate + D-alanine ↔ pyruvate + D-glutamate; (B) α-ketoglutarate + D-leucine ↔ D-glutamate + 4-metyl-2-oxovalerate; (C) 4-metyl-2-oxovalerate + D-alanine ↔ D-leucine + pyruvate; (D) pyruvate + D-glutamate ↔ D-alanine + α-ketoglutarate.

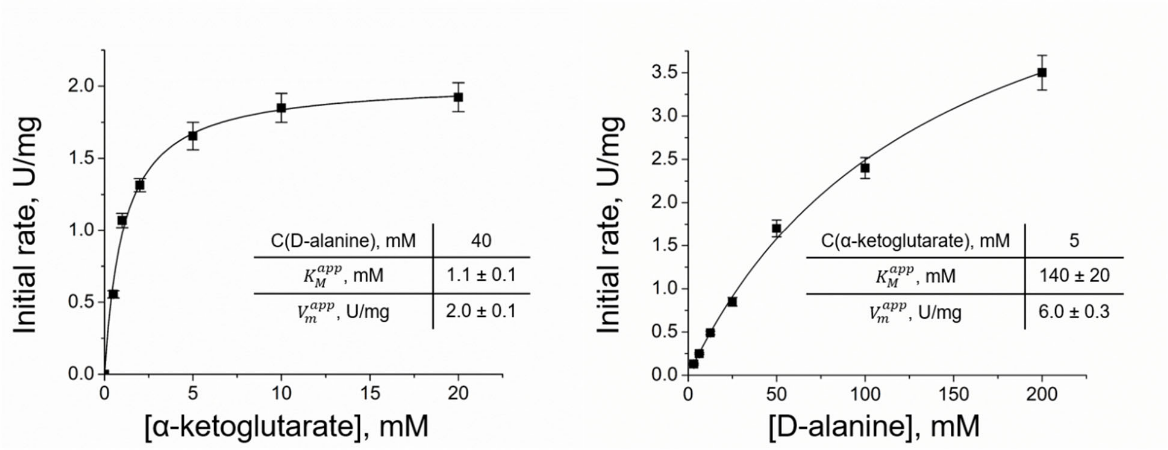

Figure S7. Steady-state kinetics of the transamination reaction catalyzed by the R179G variant. Reaction: α-ketoglutarate + D-alanine ↔ pyruvate + D-glutamate.

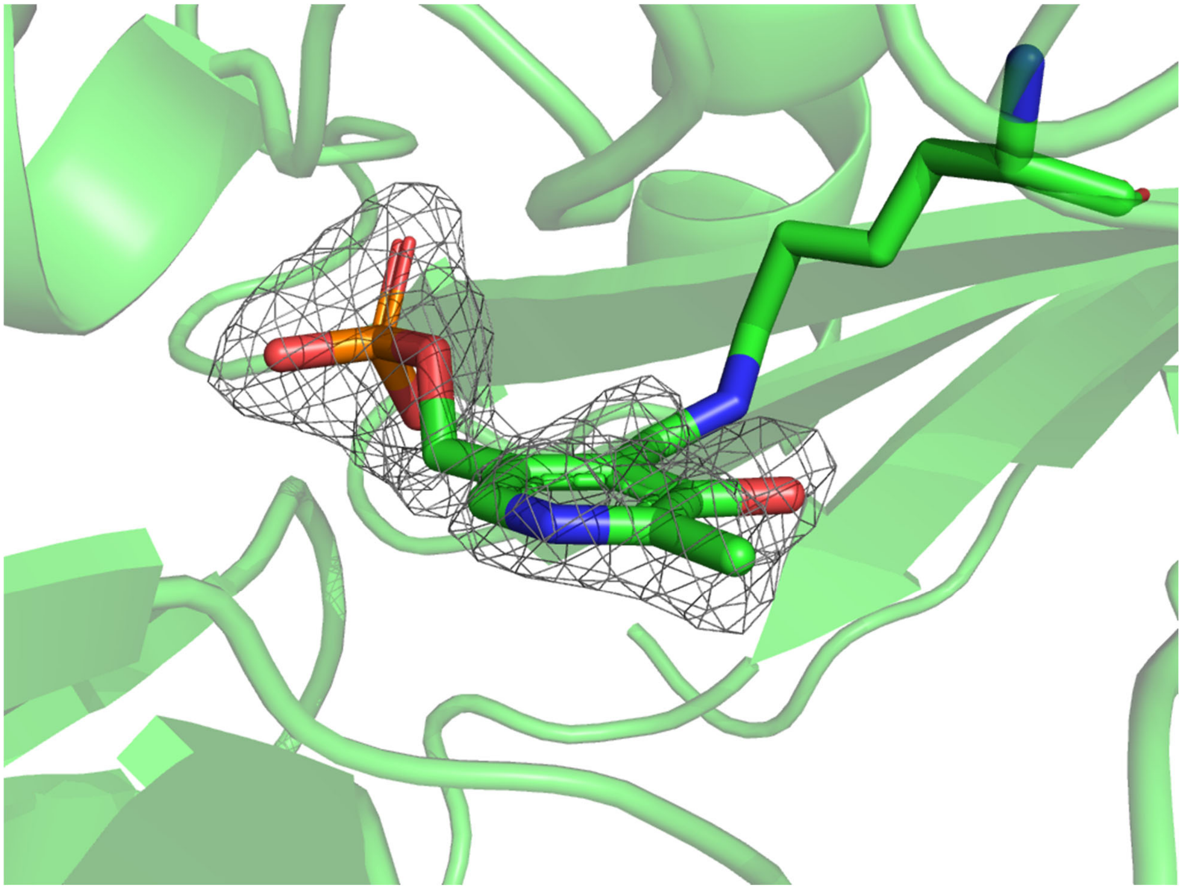

Figure S8. Omit density map for the PLP molecule in Halhy holo structure. Density map is shown as a gray mesh at the 3σ level. Protein is shown as a semi-transparent cartoon. The lysine residue covalently bound to PLP is also depicted.

References

1. Bae, H.-S.; Lee, S.-G.; Hong, S.-P.; Kwak, M.-S.; Esaki, N.; Soda, K.; Sung, M.-H. Production of aromatic D-amino acids from α-keto acids and ammonia by coupling of four enzyme reactions. *J. Mol. Catal. B Enzym.* **1999**, *6*, 241–247, doi:10.1016/S1381-1177(98)00073-3.
